# ChromCall: assigning chromatin status to defined genomic regions using epigenomic profiling data

**DOI:** 10.1093/bioinformatics/btag336

**Published:** 2026-05-24

**Authors:** Bo Wang, Muna Al-Jabri, Udayaraja GK, Alastair Droop, Lucy F Stead

**Affiliations:** University of Leeds, Leeds, LS9 7TF, United Kingdom; University of Leeds, Leeds, LS9 7TF, United Kingdom; University of Leeds, Leeds, LS9 7TF, United Kingdom; Aarhus University, Aarhus, 8000, Denmark; University of York, York, YO10 5DD, United Kingdom; University of Leeds, Leeds, LS9 7TF, United Kingdom

## Abstract

**Motivation:**

Chromatin regulation is crucial for modulating gene expression and cellular function by altering DNA accessibility. Defining and understanding chromatin regulation across diverse biological conditions, including health and disease, requires quantification of both the presence and enrichment level of diverse DNA-binding factors and chromatin modifications across defined genomic regions. Existing approaches mainly rely on peak-based or genome-wide models, which identify high-signal regions but do not annotate chromatin status at predefined functional genomic regions, such as promoters or enhancers. This lack of region-based annotation limits downstream comparative and integrative analyses across multiple factors and datasets, prompting us to create ChromCall.

**Results:**

ChromCall is an R package for region-based chromatin enrichment analysis that provides a robust and extensible foundation for transparent and reproducible epigenomic profiling at predefined genomic regions. We applied ChromCall to ChIP-seq data from glioblastoma (GBM) brain tumours and found that the promoters of genes implicated in treatment resistance are significantly more likely to exhibit a combination of histone marks associated with phenotypic plasticity. This highlights a potential novel mechanism of therapeutic escape in these deadly tumours.

**Availability and Implementation:**

The R package is available on https://github.com/GliomaGenomics/ChromCall and the version used in this paper is archived at https://doi.org/10.5281/zenodo.19580967

## 1 Introduction

Chromatin modifications and DNA-binding factors play central roles in transcriptional regulation, and their genomic distributions provide key insights into regulatory mechanisms across cell states, developmental contexts, and disease settings. High-throughput epigenomic assays such as ChIP-seq, CUT&RUN, CUT&Tag, and ATAC-seq are routinely used to profile histone marks, chromatin-associated proteins, and chromatin accessibility landscapes. In many applications, interest centres on quantifying enrichment within predefined regulatory elements such as promoters, enhancers, or transcription factor binding sites, where changes may be subtle rather than limited to the emergence or loss of canonical peaks (Mikkelsen *et al.* 2007, 2010, Rheinbay *et al.* 2013, Chatterjee and Ahituv 2017, Andersson and Sandelin 2020, Tafessu and Banaszynski 2020). This is particularly relevant when investigating signalling perturbations, therapeutic responses, or fine-grained transcriptional shifts, where small but consistent alterations in promoter- or enhancer-associated combinatorial chromatin states, defined by histone modifications and chromatin accessibility, can carry functional significance. Furthermore, determining chromatin status within consistent functional regions enables comparison between cell types, developmental stages, and disease states in biologically meaningful ways. Therefore, methods capable of sensitive, interpretable region-level quantification are essential for characterizing chromatin regulation beyond simple peak presence or absence.

However, most computational workflows remain oriented toward peak detection and genome-wide signal profiling rather than quantitative analysis within predefined regulatory regions. Peak callers effectively identify high-signal loci but yield data-dependent and experiment-specific boundaries, complicating direct region-matched comparisons across conditions (Feng *et al.* 2012, Meers *et al.* 2019). Differential enrichment tools and genome-wide binning approaches often operate on peak sets or tiled windows and frequently rely on complex statistical frameworks, limiting transparency and interpretability of sample-specific enrichment estimates (Lun and Smyth 2016, Stark and Brown 2024). Moreover, background signal can vary locally due to both technical and biological factors, yet many pipelines do not incorporate matched control data into an explicit statistical model leading to ad hoc normalization and reduced biological interpretability (Ernst and Kellis, 2012, 2017, Ramirez *et al.* 2014). As a result, researchers seeking reproducible, region-centric chromatin quantification, including promoter-level comparisons across treatments or differentiation stages, lack streamlined frameworks that unify background estimation, statistical testing, and multi-experiment comparison.

To address these limitations, we developed ChromCall, a unified, region-centred framework for transparent and statistically principled quantification of chromatin enrichment. This enables biologically interpretable, reproducible quantification of subtle chromatin changes within predefined regulatory elements and supports both single-sample assessment and two-sample comparison within a consistent statistical model. We applied ChromCall to epigenomic profiling data from glioblastoma (GBM) brain tumours. We found that the promoters of genes whose expression changes during the acquisition of treatment resistance are more likely to harbour a specific combination of histone marks. This combination is associated with the maintenance of phenotypic plasticity in stem cells, potentially highlighting a previously unknown candidate mechanism that may enable tumours to adapt and survive.

In summary, ChromCall offers a concise, transparent, and extensible solution for interrogating chromatin status, and changes therein, in specific regulatory elements. This enables biologically interpretable comparisons within and between samples.

## 2 Methods

### 2.1 ChromCall overview

**Figure 1:**
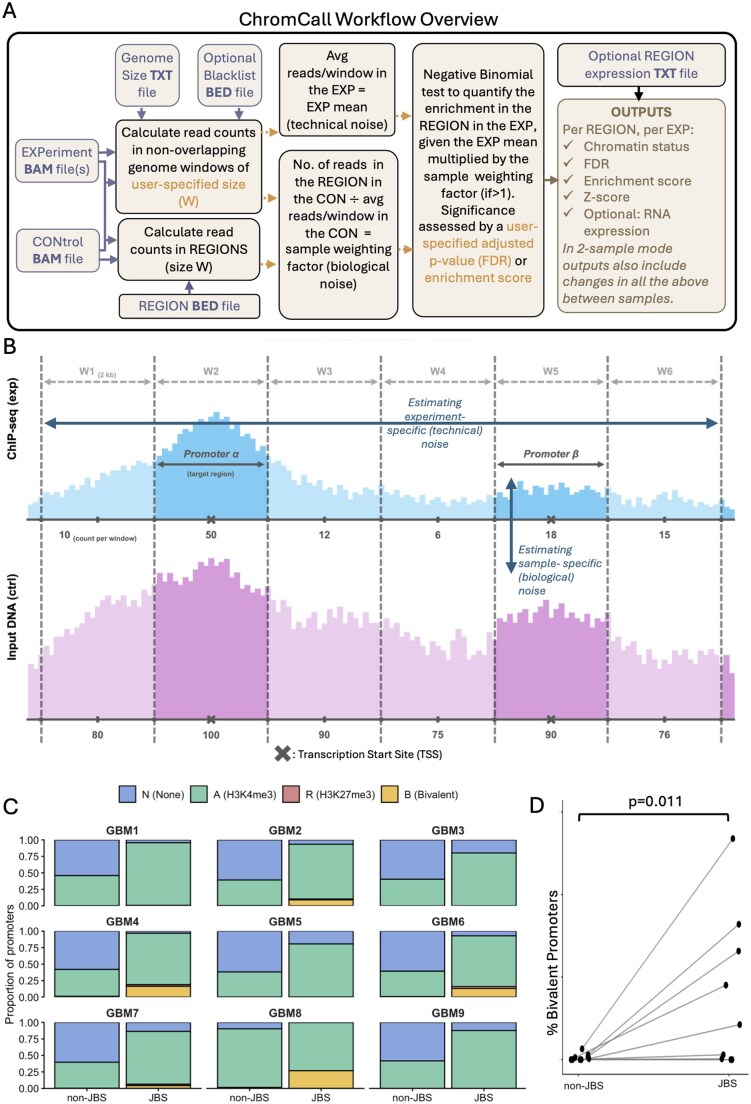
(A) Overview of the ChromCall workflow. Input files are shown in orange boxes. User-defined parameters are indicated by red text. Calculations are shown in green boxes and outputs are shown in purple box. (B) Schematic illustrating how read coverage (y-axis) from each experiment (top—blue) and its associated control (bottom—pink) is quantified across genomic windows (W) of fixed width. This demonstrates that window width is fixed and matches the predefined genomic regions of interest; in this example, promoters defined as 1 kb upstream and downstream of the transcription start site (marked by an X). Enrichment calculations account for technical variation using experiment-specific genome-wide binding averages and for biological variation by assessing enrichment at the same region in a matched sample control. (C) Proportion of non-JBSgene and JBSgene promoters assigned to different chromatin states, as determined by applying ChromCall to ChIP-seq data from nine primary glioblastoma (GBM) samples. (D) Paired dotplot showing the percentage of bivalent status calls in non-JBS vs JBS promoters for all of the 9 GBM samples in part C.

ChromCall is an R package that quantifies the enrichment of chromatin profiling signals across predefined genomic windows, such as promoters, from epigenomic next-generation sequencing alignment data (e.g., ChIP-seq, CUT&RUN, or ATAC-seq). Figure 1A gives an overview of the approach, in which read alignment and count enrichment in each region are ascertained against a background of experiment-specific (technical) and sample-specific (biological) noise. ChromCall performs region-wise statistical inference using a unified global background framework, with or without an associated control, enabling consistent enrichment estimation across datasets. ChromCall outputs region-level chromatin status, indicating whether the epigenomic profile is absent 0 or present (1), along with false discovery rate (FDR)-adjusted significance measures, and both an intuitive enrichment score and a z-score that capture the magnitude and statistical strength of enrichment, respectively, facilitating ranking and visualization. Users have the option to provide RNA expression data associated with the region of interest, which is then integrated into the output file to enable downstream integrative analyses. Beyond single-sample analyses, ChromCall enables pairwise comparisons to identify region-level differences between two samples, providing delta metrics, such as changes in enrichment and z-score, to quantify chromatin pattern differences and associated changes in expression (where supplied for each sample). Results are organized into integrated data structures with associated comparisons and metadata, enabling straightforward export and downstream analysis of region-level chromatin enrichment.

### 2.2 Background estimation and local modulation

ChromCall quantifies chromatin enrichment by summarising aligned sequencing fragments within predefined genomic regions (Fig. 1B). Paired-end reads are combined into fragments spanning the mapped coordinates for each pair, while single-end reads are treated as individual fragments. To ensure consistent quantification across paired- and single-end datasets with varying fragment lengths, each fragment is represented by its central genomic position, corresponding to the midpoint of the sequenced DNA molecule. For each experiment and its associated control (if provided), fragment centres falling within predefined genomic windows are counted to obtain region-level read counts. These counts form the empirical basis for background estimation and subsequent enrichment inference.

ChromCall models read counts using a Negative Binomial framework, which accommodates overdispersion commonly observed in sequencing-based chromatin profiling data, and has previously been shown to be optimal for these types of data (Cairns *et al.* 2014). The model is parameterised by a genome-wide background mean and a global dispersion parameter estimated from genome-wide tiles, enabling robust region-level inference without requiring replicate-level dispersion estimation.

To establish a global baseline for each experiment, ChromCall estimates a genome-wide background rate $\lambda_{g}$ as the mean read count per genomic tile, excluding blacklisted regions:


$$\lambda_{g}=\frac{1}{N}\sum_{i=1}^{N}y_{i},$$

where $y_{i}$ is the read count in the $i^{th}$ non-blacklisted tile and N is the total number of such tiles. Zero-count tiles are retained by default to avoid upward bias in sparse datasets, ensuring that $\lambda_{g}$ reflects the overall sequencing background rather than local enrichment.

While the genome-wide background rate provides a useful global reference, it cannot fully account for sample-specific regional differences arising from chromatin accessibility, copy-number variation, or sequencing bias. To correct for such local variability, ChromCall uses a control-based modulation factor for each region $m_{i}$, derived from the control experiment (when available) as


$$m_{i}=\max(1,\frac{y_{i}^{\left(\mathrm{ctrl}\right)}}{\lambda_{g}^{\left(\mathrm{ctrl}\right)}}),$$

where $y_{i}^{\left(\mathrm{ctrl}\right)}$ is the observed read count in the same region in the control and $\lambda_{g}^{\left(\mathrm{ctrl}\right)}$ is the corresponding genome-wide background rate. The lower bound of one prevents deflation in regions with low control signal. The expected signal for region i in experiment j is then expressed as


$$\lambda_{t,i}^{\left(j\right)}=m_{i}\cdot \lambda_{g}^{\left(j\right)},$$

where $\lambda_{t,i}^{\left(j\right)}$ represents the locally adjusted expected background that integrates global background intensity with region-specific modulation. This formulation preserves statistical transparency while accounting for both global sequencing depth and local control variation, providing a stable foundation for subsequent enrichment testing. In the absence of a matched control (e.g., ATAC-seq), $m_{i}$ is set to 1, and inference is performed relative to the global background model alone. Together, these steps establish a unified and interpretable baseline model that enables consistent region-level inference across experiments.

### 2.3 Statistical testing and enrichment scoring

ChromCall performs region-wise hypothesis testing to evaluate whether the observed read counts in each region in each experiment significantly exceed the locally adjusted expected background derived from the background model. For each region i and experiment j, the observed count $y_{i}^{\left(j\right)}$ is compared against its expected value $\lambda_{t,i}^{\left(j\right)}$ using a one-sided Negative Binomial test to assess enrichment:


$$p_{i}^{\left(j\right)}=P(Y\geq y_{i}^{\left(j\right)}|Y∼\mathrm{NB}(\mu =\lambda_{t,i}^{\left(j\right)},\mathrm{size}=\theta^{\left(j\right)}))$$

where $\theta^{\left(j\right)}$ denotes the global dispersion parameter estimated for experiment j. The dispersion parameter is estimated from genome-wide background tiles and shared across regions within each experiment, reflecting the assumption that overdispersion primarily arises from technical variability in sequencing and library preparation, which is consistent across genomic regions within a sample. In the absence of replicates, this global estimation provides a stable and robust approximation, avoiding unreliable region-specific dispersion estimates. In cases where the estimated dispersion approaches infinity, the model reduces to a Poisson distribution. Multiple testing correction is applied across all regions using the Benjamini–Hochberg false discovery rate (FDR) procedure to obtain adjusted *P*value.

In addition to significance testing, ChromCall reports additional quantitative metrics that describe both the magnitude and statistical strength of enrichment. The enrichment score for each region is defined as the log ratio between observed and expected counts:


$$s_{i}^{\left(j\right)}=\log_{2}\left(\frac{y_{i}^{\left(j\right)}+\epsilon }{\lambda_{t,i}^{\left(j\right)}+\epsilon }\right)$$

where ϵ is a small pseudo-count added for numerical stability. To quantify the standardised deviation of observed counts from the expected background under the Negative Binomial model, a z-score is computed as


$$z_{i}^{\left(j\right)}=\frac{y_{i}^{\left(j\right)}-\lambda_{t,i}^{\left(j\right)}}{\sqrt{\lambda_{t,i}^{\left(j\right)}+\frac{(\lambda_{t,i}^{\left(j\right)}{)}^{2}}{\theta^{\left(j\right)}}}}\frac{y_{i}^{\left(j\right)}-\lambda_{t,i}^{\left(j\right)}}{\sqrt{\lambda_{t,i}^{\left(j\right)}}}$$

Together, these statistics, FDR-adjusted *P*value, enrichment scores, and z-scores, provide complementary measures of chromatin signal variation, supporting both formal statistical inference and effect-size–based prioritization of enriched regions across experiments.

### 2.4 Target region delineation and expression

ChromCall operates on fixed genomic windows that define the units of quantification. The default supplied regions are human promoters, defined as 2 kb windows spanning 1 kb upstream and 1 kb downstream of the transcription start site (TSS). Users may alternatively supply custom predefined genomic regions, however, these regions must be fixed width W, which is specified as an input parameter. We recommend that target regions be independent of the histone marks being tested, to avoid circularity between region definition and ChromCall enrichment calling.

An optional genome blacklist file may also be provided, detailing regions to be excluded from downstream analyses. Regions overlapping blacklisted intervals are flagged and can be excluded during background estimation and statistical testing. An optional control dataset may also be supplied to model background signal; in its absence, ChromCall relies on genome-wide background estimation from the target data alone. Region-based expression measurements can be incorporated by mapping gene-level expression values to genomic windows based on transcription start site (TSS) annotation, and appending them to the region metadata, enabling joint interrogation of chromatin enrichment and transcriptional output without requiring additional preprocessing steps.

These annotation layers remain optional and non-intrusive, allowing users to perform chromatin-only analyses or to integrate transcriptional data where relevant while retaining full control over normalization and modelling choices. This modular architecture also facilitates future extensions to additional statistical models or multi-sample designs.

### 2.5 Application to glioblastoma ChIP-seq data

ChlP-seq data for the GBM datasets were acquired as fastq files. Reads were subjected to quality control using FastQC, followed by adapter and quality trimming with Trim Galore in paired-end mode, retaining reads with a minimum Phred quality score of 25 and a minimum post-trimming length of 20 bp. Trimmed reads were aligned to the GRCh38 primary assembly reference genome using Bowtie2 in end-to-end, very-sensitive mode, with mixed and discordant alignments disabled and insert sizes constrained between 10 bp and 1000 bp. Alignments were coordinate-sorted and assigned read group information during BAM generation. Alignments were filtered to retain only properly paired reads while excluding unmapped reads, reads with unmapped mates, and secondary alignments. PCR duplicates were removed using Picard, and reads with low alignment confidence were excluded by retaining only alignments with a mapping quality of at least 20. Final BAM files were indexed using samtools.

Region-level chromatin enrichment analysis was performed using ChromCall. For each biological sample, enrichment was quantified for histone marks H3K27me3 and H3K4me3 relative to a matched input control, using a GRCh38 genome size reference derived from the GENCODE v48 primary assembly annotation, a predefined promoter region set derived from the GENCODE v48 annotation provided as a BED file, a fixed window size of 2000 bp, and the ENCODE hg38 genome blacklist to exclude problematic genomic regions. A histone mark was classified as present at a promoter when the enrichment score exceeded 1.5 and the Benjamini-Hochberg false discovery rate-adjusted *P* value was less than 0.25. This FDR threshold of 0.25 was chosen, consistent with prior region-based chromatin enrichment studies, as it balances sensitivity and specificity for broad promoter-associated marks. Importantly, our primary analyses rely on *comparative proportions* (e.g. comparing promoters containing a JARID2 binding site, versus those that don’t), which are robust to this threshold choice.

## 3 Application

The logic employed in ChromCall to detect specific genomic regions of enriched epigenomic profiling signal has been applied in multiple studies across different biomedical domains (Mikkelsen *et al.* 2007, 2010, Rheinbay *et al.* 2013), but has not previously been packaged into an accessible, user-friendly software tool. We have previously, specifically applied the ChromCall approach to epigenomic profiling data from longitudinal (primary and post-treatment recurrent) glioblastoma (GBM) brain tumours as follows (Tanner *et al.* 2024). Gene expression analysis of longitudinal GBM samples previously identified a subset of genes that significantly and consistently change expression through treatment, suggesting their potential involvement in therapeutic resistance (Tanner *et al.* 2024). This is subset is denoted JBSgenes, as the promoters all contain JARID2 binding sites (JBS). JARID2 is an accessory subunit for Polycomb Repressive Complex 2 (PRC2), which is responsible for the deposition of the repressive histone mark H3K27me3. Our previous study, therefore, used the ChromCall approach to investigate the H3K27me3 status of JBSgene promoters in GBM samples, and found significant enrichment compared with the promoters of non-JBSgenes (Tanner *et al.* 2024).

Since then, we have developed and packaged ChromCall to formalise this region-based enrichment workflow into a transparent and reusable framework capable of calling multiple epigenetic marks across specific genomic regions. To extend our analysis, we additionally investigated H3K4me3 promoter status using datasets acquired from the same nine primary GBM samples where we previously investigated H3K27me3 (Hall *et al.* 2018, Tanner *et al.* 2024). Using the integrated H3K4me3 and H3K27me3 outputs from ChromCall, promoters were assigned to four discrete chromatin states: N (neither mark), A (active; H3K4me3 only), R (repressed; H3K27me3 only), and B (bivalent; both marks). Promoters were subsequently partitioned into JBSgene and non-JBSgene sets.

Our results (Fig. 1C) show that H3K4me3 is also significantly enriched at JBSgene promoters and that, importantly, enrichment of H3K27me3 co-occurs with H3K4me3, indicating that JBSgene promoters are significantly (paired 1-sided Wilcoxon test: *P* = 0.011) more likely to be bivalent than non-JBSgene promoters (Fig. 1D). These results were robust to alternative promoter definitions [TSS ±1 kb, 2 kb, or 5 kb (Tanner *et al.* 2024)] and consistent across samples (Fig. 1C). Bivalency was first observed at lineage-determining genes in embryonic stem (ES) cells, where subsequent resolution to a single histone mark was shown to lead to cell fate commitment (Bernstein *et al.* 2006, Mikkelsen *et al.* 2007). Conversely, retention of bivalency maintains developmental potential, conferring phenotypic plasticity and the ability to respond to external cues. Importantly, the subsequent multi-lineage differentiation capability of ES cells has been shown to require transcriptional priming via a JARID2-dependent mechanism (Landeira *et al.* 2010). Our results suggest that JBSgene promoters are bivalent in GBM tumours, which may facilitate the tumour’s ability to rapidly adapt to, and survive, chemoradiation. This mechanism of phenotypic plasticity warrants further investigation in GBM as a potential therapeutic vulnerability.

## 4 Conclusion

ChromCall integrates all steps required for region-based epigenomic profiling enrichment analysis, including ChIP-seq, CUT&RUN, CUT&Tag, and, ATAC-seq, within a single reproducible framework. By removing the need for bespoke code, ChromCall ensures that identical analytical procedures are applied consistently across samples, experiments, and genomic region sets. The workflow is fully extensible, enabling joint analysis of multiple epigenomic profiles, while applying the same analytical structure to promoters, enhancers, super-enhancers, differential peak sets, or user-defined genomic intervals without modification. This flexibility establishes ChromCall as a generalisable and transparent framework for multi-mark, region-based enrichment analysis across diverse epigenomic contexts.

Application of ChromCall to publicly available GBM ChIP-seq data highlights its ability to perform promoter-level enrichment scoring for multiple histone marks, integrate mark-specific signals into interpretable chromatin-state classifications, and facilitate reproducible comparisons between biologically defined promoter groups. Together, these features enable robust region-centric interrogation of chromatin regulation and support the discovery of biologically meaningful patterns in complex disease settings.

## Author contributions

Wang Bo [Data Curation (Supporting), Formal Analysis (Lead), Investigation (Supporting), Methodology (Supporting), Software (Supporting), Validation (Lead), Visualization (Lead), Writing Original Draft (Lead), Writing Review Editing (Equal)], Al-Jabri Muna [Formal Analysis (Supporting), Investigation (Supporting), Validation (Supporting), Writing Review Editing (Equal)], GK Udayaraja [Formal Analysis (Supporting), Investigation (Supporting), Software (Supporting), Writing Review Editing (Equal)], Droop Alastair [Software (Lead), Writing Review Editing (Equal)], and Stead F. Lucy [Conceptualization (Lead), Data Curation (Lead), Formal Analysis (Supporting), Funding Acquisition (Lead), Investigation (Lead), Methodology (Lead), Project Administration (Lead), Software (Supporting), Supervision (Lead), Validation (Supporting), Writing Original Draft (Supporting), Writing Review Editing (Equal)]

## Data Availability

ChIPseq data from primary GBM tumors was acquired from the Sequencing Read Archive (https://www.ncbi.nlm.nih.gov/bioproject/PRJNA391756) (Hall *et al.* 2018).
